# Modeling and Analyzing the Availability of Technical Professional Profiles for the Success of Smart Cities Projects in Europe

**DOI:** 10.3390/s24186089

**Published:** 2024-09-20

**Authors:** Inés López-Baldominos, Vera Pospelova, Luis Fernández-Sanz, Ana Castillo-Martínez

**Affiliations:** Department of Computer Sciences, Universidad de Alcalá, E-28805 Alcalá de Henares, Spain; ines.lopezb@uah.es (I.L.-B.); vera.pospelova@uah.es (V.P.); ana.castillo@uah.es (A.C.-M.)

**Keywords:** smart cities, human factors, Europe, qualification, availability

## Abstract

The success of developing and implementing Smart Cities (SC) projects depends on a varied set of factors, where the availability of a qualified technical workforce is a critical one. The combination of ICT requirements, like the effectiveness and quality of solutions merging IoT, cloud computing, sensors, and communications with the work from many varied disciplines (e.g., civil engineering, architecture, etc.), mixed with aspects of environmental and business sustainability, makes the management of these projects really challenging. Reports forecast a scarcity of qualified candidates, given this complexity and the growth of activity in SC projects. The European project SMACITE has addressed the requirements of the qualification of an ICT workforce with an analysis of multiples sources of information from the labor market, feedback from involved stakeholders, and the literature. The goal was the development of two occupational ICT profiles as a reference for training and for the availability of candidates for job vacancies. The result is two ICT role profiles for engineers and technicians, mapped with the European skills frameworks ESCO and EN16234. The profiles determined the whole set of requirements, including not only the technical areas and soft skills, but also additional technical areas and sustainability and managerial skills and the analysis of different sources of information. Our work has also determined which existing ESCO occupations are similar to the two reference profiles, so they are better adapted to SC projects. The training activities of SMACITE have also suggested the amount of training expected for a varied sample of candidates who want to be qualified for SC projects.

## 1. Introduction

A Smart City (SC) is a city where traditional infrastructures and services become more efficient and effective by exploiting digital solutions for the benefit of citizens and businesses. The benefits do not only refer to the optimization of resources and enhancement of services, rather they indirectly have a positive impact on sustainability understood as an improved quality of life, which can be defined as follows: “meeting the needs of the present without compromising the ability of future generations to meet their own needs” [1,2]. Sustainable development is a priority objective for the European Union’s internal and external policies [3], also aligned to the set of 17 sustainable development goals of the United Nations [4], especially contributing to SDG 11 for sustainable cities [5,6] or SGD 7 for clean energy [7,8].

The prospective analysis on the global market for Smart Cities shows how, in 2019, the market was valued at USD 392.9bn [9], but it is predicted to grow as high as USD 3.7trn by 2030 [10]. This impulse will lead to a higher demand for the provision of technical and digital services, where qualified human resources play a key role in the success of the implementation of projects for SC. Maturity models for the implementation of SC have become popular in recent years, with a good number of those already available (see a review in [11]) addressing the key factors for the success of projects. These models have been sharing aspects and a parallel development with maturity models for effective IoT implementation, as IoT is identified as a key technology for SC (see, e.g., [12]) or even a pre-requisite for maturity in SC projects [13]. Klisenko and Serral [14] analyzed 16 different maturity models applicable to the readiness for IoT and have identified the capabilities of the IoT implementation support team as an essential resource for success, also identified in specific projects [15,16]. Research [17] shows other references to the key role of qualified technical personnel in projects. In the specific case of SC projects, one of the major organizational risks is the unavailability of the required human resources, as these projects are based on innovative ideas and require highly skilled human resources to be completed [18]; therefore, facilitating and developing training capabilities is needed to mitigate such risk. Although the impact of the availability of qualified human resources is not restricted to technical personnel [19], the development of human resources with the necessary technical skills is essential to provide solutions, correct management, and technology at different levels in a Smart City [20].

This research is aimed at studying the recommended professional profiles and the sources of availability of qualified ICT personnel, as both are identified as key factors for the success of SC projects. The first goal is the modeling of two ICT role profiles for engineers and for technicians in a way that enables a clear understanding of the recommended set of functions, skills, and knowledge using the following main skill frameworks in the EU: ESCO, the official EU labor classification, and the standard EN16234 [21]. Using these common references will allow for a quick and easy understanding of the terminology and concepts used in the profiles. Once the profiles are ready, the second goal is the analysis of possible sources of availability of personnel for both roles. One source of candidates for populating the workforce for these ICT profiles for SC projects is the analysis of those existing professional profiles from ESCO that are closer to the recommended ones for SC engineers and technicians, so they would need less adaptation for effective work in projects. Another analysis for the availability of candidates is based on checking if a sample of people interested in the work in SC already have, before any specific training, the skills and the knowledge of the technical areas recommended for SC engineers and technicians.

Therefore, this study started from the initial technical professional profiles for IoT projects determined through the data analysis of the SMACITE project, a project focused on the supply of professionals for SC projects. Although IoT is a key factor for SC projects, this study expands the profiles determined for IoT projects with additional, specific aspects linked to SC projects like cloud computing, green, or managerial skills, which have already been considered in the study of [22]. Once the key aspects of the recommended profiles were determined, this study addresses different sources of information to determine the options for the supply of technical SC professionals, analyzing possible similar profiles in the labor market, thanks to the standardization of terminology from the ESCO labor classification. The information from specific training experiences for SC profiles will also provide additional insights into the options for supplying enough SC technical profiles for the expected demand of SC projects.

This article is structured as follows: Section 2 presents the research questions of our study and the methodology for answering them, briefly defining the motivation and goals of the different mechanisms for collecting and analyzing information. Section 3 presents and discusses the recommended professional profiles, expanded from the reference of IoT projects to cover all aspects of SC projects. Section 4 describes the possible supply of similar profiles to the ones recommended for SC projects according to the analysis of the most relevant on the ESCO labor classification and the standard EN16234, the most relevant skills frameworks in the EU. Section 5 describes and discusses the experience of diagnostics of the capacities and training courses implemented by the SMACITE project as a complement for increasing the availability of qualified technical personnel. Section 6 is a discussion of the results and the relation to research questions. Finally, Section 7 proposes conclusions and future research lines.

## 2. Methodology

Before describing the process and methodology followed for this work, it is important to clarify that, given the multidisciplinary scope of SC projects, our approach adopts the paradigm concerning the engineering/computer science expressed in [23]. Therefore, the focus will be on the interaction between different types of data and the three pillars of SC (i.e., infrastructures, processes, and people) representing the basis for providing services to several application domains. The ICT roles will be mainly involved in the integration and exploitation of subsystems, including the development of support for big data and decision making. Moreover, the SMACITE project decided to work with two potential qualification profiles, given the two main occupation groups for ICT in the ESCO labor classification, being ICT professionals/engineers (group 25 of ESCO) and ICT technicians (group 35 of ESCO). The project did not address the other relevant group of ICT occupations corresponding to ICT managers (group 1330 of ESCO). In the case of IT managers, we can refer to the ICT role profiles developed as part of the SmartDevOps project, which was considered as the first basis for SMACITE (albeit with a totally different approach, given it focuses on DevOps [24]). In [25,26], we can find references to a Smart City IT manager and a Smart City IT officer.

After clarifying the target of the roles and occupations to be studied, the process and methodology for our work is logically determined by the main goals already declared. The process is then guided by the following research questions as an expression of our research goals:

- RQ1: Which is the most recommended qualification profile for ICT engineers and technicians in Europe for an effective implementation of SC projects?
- RQ2: Which occupation profiles in the ESCO labor classification are closest to the recommended qualification profile for SC projects?
- RQ3: Which is the preliminary qualification in the technical areas of a sample of candidates to a training program for the SC-recommended profiles?

**Figure 1:**
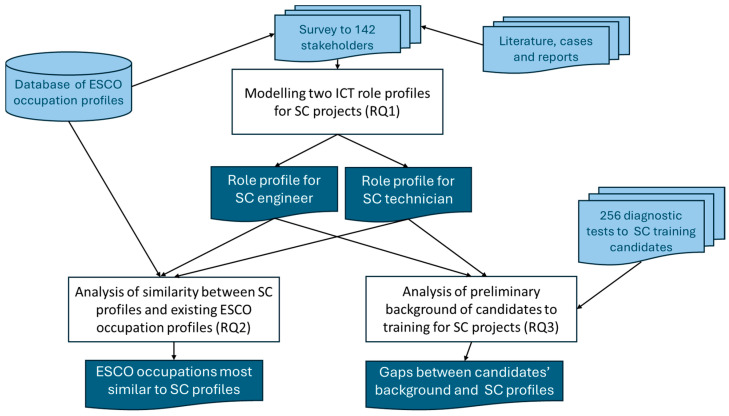
Methodology and sources of information used to solve the proposed RQ.

This is summarized in Figure 1, with white boxes for the RQs and associated activities, while the input information is shown in light blue, and the results have a dark blue background. The first goal is the modeling of the qualification of ICT roles required by SC projects (RQ1) using the results of the SMACITE survey, developed (see [17]) with the reference of the literature, reports, and cases and expressed with items of ESCO. The two profiles served as basis for the following:

- Analyzing the availability of qualified candidates through the study of the similarity with existing ESCO occupation profiles (RQ2): the more similar an ESCO occupation is to the recommended SC profiles, the easier the adaptation of those professionals to SC projects.
- Forecasting the expected effort of training for the technical areas of the modeled profiles by studying the results of diagnostic test of candidates to training in SC technical areas and comparing them to the expected SC profiles: the more skills and knowledge already available in candidates, the less effort is needed to get them qualified in those SC technical areas.

As briefly commented above, the main method for answering RQ1 requires the exploitation of extra data collected within the frame of the SMACITE projects, an EU-funded project on SC projects that explores the recommended professional profiles in this area. A first report of the project was already used for the analysis of the qualification of the technical team in this part of IoT in SC projects [17]. The main instrument for the collection of data was a survey to collect the opinion of a broad base of stakeholders to determine the recommended skills and knowledge profile for ICT professionals working in the context of SC projects, both at the engineer and technician level. As already explained in [17], the mapping of the two most relevant EU frameworks for technical occupations and job roles was a key element to ensure the best applicability across Europe, as it follows common references like ESCO [27]; the European multilingual classification of skills, competences, qualifications, and occupations and EN16234 [21], the European standard on e-competences for ICT professionals. The survey was designed to collect the technical aspects, such as the IoT technology, cybersecurity, data analytics, and machine learning (recommended e.g., by [28] and already treated in [17]), but also other relevant areas for SC projects, such as cloud computing (recommended in e.g., [29]). The recommended soft skills (non-technical skills) were already considered in the first survey for IoT projects, as they are linked to success in effectiveness, professional performance, and career [30,31], confirmed by employers, as they consider that the performance of a hard skill is often dependent upon soft-skill capacity [32]. As there is no widely accepted model of soft skills, we adopted that of the Skills Match project [33,34], with a framework of 36 skills, also directly matched to ESCO [27].

An extended version of the results of the survey has been used for the profiles for SC projects now considering—apart from the already mentioned of area of cloud computing—the analysis of two other relevant aspects for SC projects, as follows: green/sustainability and managerial skills (especially highlighted in [35,36] and in the SLR on challenges for Smart Cities [37] as key factors for those projects). The new version of the dataset has 10 more responses and helps to consolidate the profiles that serve as a basis for the rest of the study.

As the key point for success of the SC projects is the availability of qualified technical personnel, as commented in Section 1, RQ2 focused on the comparison of the requirements for the recommended profiles to the official profiles of occupations of the ESCO labor classification. Given the large number of items—both skills and knowledge items (13939 in version 1.2) and occupations (3008 in version 1.2) of the ESCO classification—this comparison is only effective and feasible when it is possible to execute sophisticated queries on the ESCO database, something that it is not possible by the simple consultation of the classification through the public website. This analysis is only possible thanks to the local replication of the whole ESCO database that we made to enable this type of query. The results extracted from the database will provide insights into the occupational profiles of ESCO that are closer to the requirements for SC profiles, so they can define which initial technical profiles can be more easily uptrained to best fit the recommended profiles for SC projects. Finally, the answer to RQ2 contributes to the depiction of the perspective of possible sources of similar profiles that could be urgently trained to supply enough tech talent for the expected growth of SC projects and implementations [38].

However, it is not always possible to find candidates with those profiles for specific training on the recommended knowledge and skills for SC projects. The SMACITE project has developed a set of preliminary assessment and diagnostic tools for the prospective students of the training courses generated as part of the project. RQ3 will use the results of such diagnostics from 86 trainees enrolled in SMACITE training course to provide insights on their qualification in the main technical areas considered in the recommended SC profiles and its influence on training completion. The results of this experience could result in better knowledge of the challenges for training more SC specialists, as follows: the results from this random sample of candidates interested in the specialization as SC engineers and technicians could provide a preliminary, although obviously limited, idea of the expected command of the technical areas of SC among potential new specialists before starting training. The detected gaps in relation to the SC-recommended profiles would determine the amount of training effort required to be aligned with the needs for SC projects. In the end, the bigger the gaps, the more training effort to reach the expected profile is required.

## 3. Analysis of Recommended Technical Profiles for Successful SC Projects

As commented in Section 2, the study of the qualification of the technical team for SC projects worked with the data collected with the survey of the SMACITE project [39]. This article uses the extended dataset, which includes 10 more responses compared to the one used for the article on IoT profiles [17]. The collected information will be the basis for the answer to RQ1. As already explained in [17], the survey was targeted to the following three different categories of stakeholders linked to SC projects: (a) the customer side, with municipal authorities, managers, and technicians; (b) the provider side, with managers and professionals from solution-development companies; and (c) the user side, with representatives of citizens’ associations and independent experts. The rest of the details were similar in the two surveys.

### 3.1. Sample

The project partners disseminated the surveys through different networks, specifically targeting contacts belonging to any of the categories of stakeholders. The rate of participation was 31.6% (142 from 449 clicks on the online link). The nationality of the respondents was diverse, with 11 European countries identified in both. The highest number of contributions came from Spain (35.92%), Greece (15.49%), Bulgaria (26.06%), and Italy (12.68%). The gender representation was unbalanced, with the following data: male (70.42%), female (24.65%), and prefer not to say their gender (2.11%).

The stakeholder category included three main options, with different sub-options as job roles, as follows: public sector and authorities (client side), business sector and providers (supply side), and civil society (user side). Table 1 shows the distribution among the roles of the three categories in the two surveys, showing the variety of roles (except for the case of “Municipal city planner or urbanism expert,” which was without representation). 

The data on years of professional experience were organized into groups of five, with options for less than 5 years, steps of five between 5 and 20, and more than 20. The contributors were mostly concentrated in the segment of more than 20 years (46.48%) and less than 5 years (15.49%), while the rest of the options were under 12%. The self-declaration of participants referred to their familiarity with SC concepts and solutions on a scale with five options, and the results were as follows: none (4.93%), basic knowledge (29.58%), application of concepts out of professional practice (25.35%), professional experience in the area (27.46%), and highly qualified and experienced in the area (12.68%).

### 3.2. Analysis of Results on Qualification of the Technical Team for SC Projects

As this is an extended analysis of results to cover all aspects required by SC projects, all of the descriptions of the survey are the same as those found in [17]. The relevant results come from the main section of the survey collecting the opinion of the participants on the set of functions, skills, and knowledge based on ESCO and e-CF [21] recommended for the qualification profile, for both engineer and technician roles. As explained in [17], 15 ESCO occupations—out of a total of 91 for ICT and 89 for knowledge and skills items, from a total of 13,939 in the labor classification—were selected by an expert panel. The questions adopted a 5-option Likert scale (essential, relevant, useful, marginal, and worthless) plus a “not sure” option, with a concise and synthetic writing style (peer review and pre-tests with project partners were applied before launching the survey). In the case of the e-CF (EN16234) framework [21], the final approach with e-CF worked with the mapping of the resulting set of functions from ESCO occupations to link them to the equivalent e-competences and proficiency levels in the standard. The final set of e-competences comprised nine of them (B.6, E.2, A.6., B.4, E.8, D.7, B.3, B.1, and C.1) and different levels ranging from 1 to 4, always respecting the correspondences between the competences and the levels included in the standard.

#### 3.2.1. Functions for Engineers and Technicians

**Figure 2:**
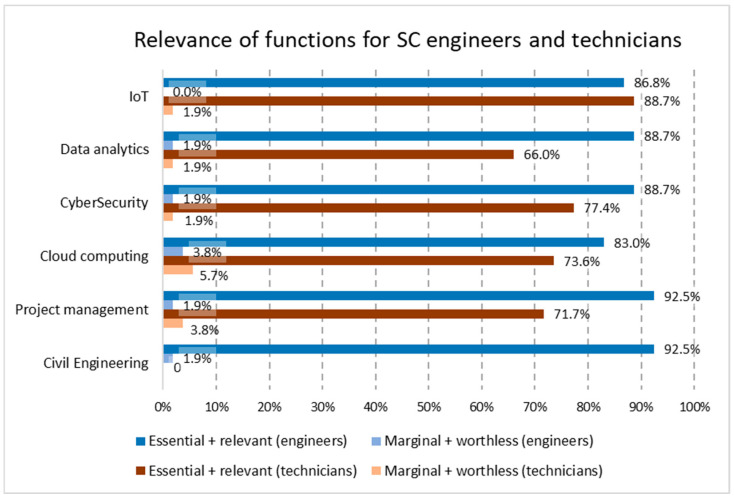
Summary of relevance of functions for SC engineers and for SC technicians.

The proposed functions for the SC engineer and the SC technician were extended to cover all of the identified aspects for SC projects, and not only IoT projects, meaning that new areas appear, especially for engineers; in addition, cloud computing, civil/construction engineering (for engineers), and project management completed the profile for SC engineers and technicians. The results from the survey show the relevance of the functions and responsibilities allocated by participants for the determination of the recommended profile of SC engineers and SC technicians (see Figure 2), grouping the percentage of those declaring essential or relevant importance and that of those opting for a value of marginal or worthless importance.

As we can see, there is less support in the results towards the assumption by technicians of functions like data analytics, project management, cybersecurity, and cloud computing. Once the profile of the functions was determined, the mapping to the EN16234 framework [21] with the equivalence of those functions with responsibilities and activities was described in dimension two of the standard for each e-competence. The final mapping developed by the expert group after analyzing the survey is shown in Table 2, and there are no changes in it. This is logical, as the differential elements in respect to the functions from the pure IoT profiles determined in [17] were embedded in the wide descriptions of the e-competences of EN16234.

#### 3.2.2. Knowledge, Skills, and Soft Skills

**Figure 3:**
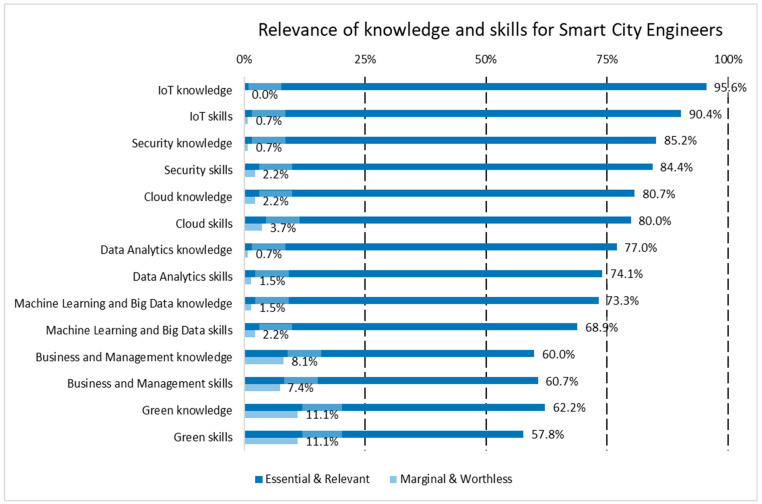
Summary of the relevance of skills and knowledge in all areas for SC engineers.

**Figure 4:**
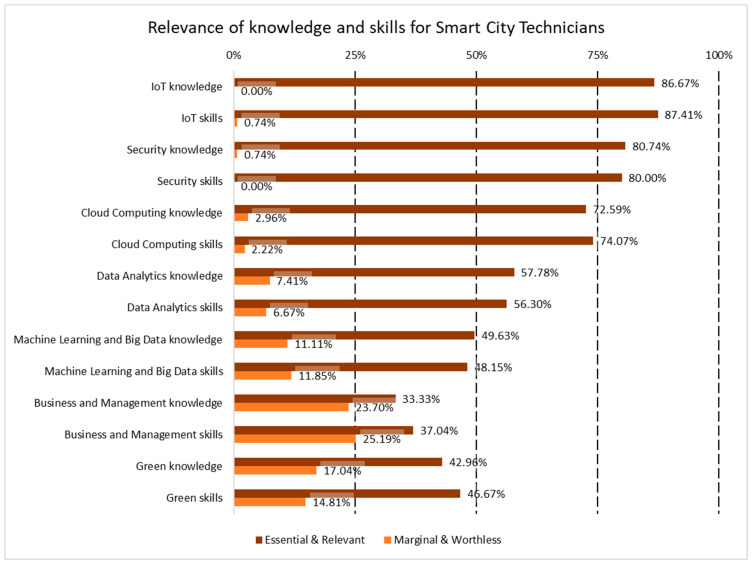
Summary of the relevance of skills and knowledge in all areas for SC technicians.

Apart from the functions to be developed by the SC engineers and technicians, the respondents to the survey gave their opinion on the relevance of the 89 skills or knowledge items identified in ESCO as being connected with SC activities. The extended version of the results of the survey does not only include the technical areas already considered in the IoT profiles and the soft skills [17], but also, as commented in Section 3.2.1, cloud computing and project management and business skills. Moreover, as commented in Section 1, the area of green skills was also analyzed, aligning the profiles to the importance of the twin transition for the EU strategy [40], while the area of civil engineering for the SC engineers was not further explored, as the focus on the details for the technical areas was on the ICT field. The profiles are shown in Figure 3 and Figure 4, showing all of the areas, technical and managerial, split into skills and knowledge.

The skills and knowledge profiles show clear differences in the areas and categories between the SC engineers and technicians. In general, the importance of knowledge over skills in each category is higher in the engineers than in the technicians, while the relevance of non-technical areas like business and management or green/environment are clearly irrelevant for technicians in the perception of the respondents to the survey. The area of machine learning and big data (both skills and knowledge) represents the least relevant option in the ranking. Again, while it is quite relevant for the engineers (around 70% for skills and knowledge), that consideration hardly reaches 50% for the technicians, therefore, this area is not considered a key factor for the qualification profile of SC technicians.

**Figure 5:**
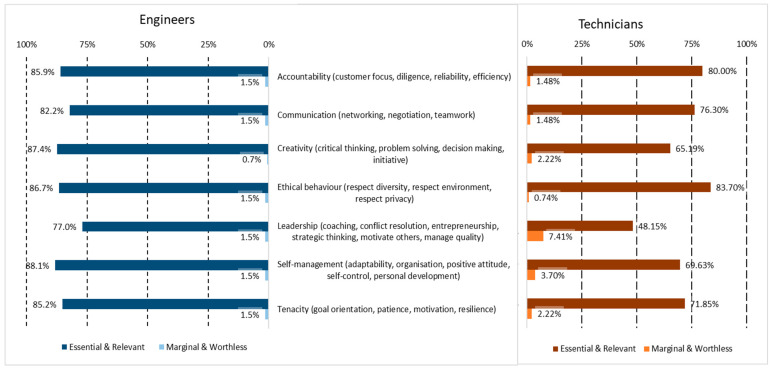
Summary of the relevance of soft skills for SC technicians.

In the case of soft skills, they were already analyzed in [17], taking the model of the Skills Match project [33,34] as a reference. The extended version of the results did not generate relevant changes in the relevance of the groups of soft skills for SC engineers, where most of the differences are below 0.5%, with highest of 0.8% in communication. However, the technicians show bigger differences, with differences close to 10% (e.g., in creativity). The final soft skills profile for both occupations is shown in Figure 5. The main difference between the engineers and the technicians is in the case of leadership, which is relevant for engineers (76.8%), while it is not for technicians (49.3%, below the threshold of 50%). Other soft skills are at a higher level of relevance in engineers, although they are considered as relatively relevant in technicians, such as creativity (88% vs. 55.6%), self-management (88% vs. 69%), and tenacity (85.2% vs. 71.8%).

The recommended profiles, as expressed in terms of the EN16234 framework and the ESCO labor classification, involve 28 knowledge items and 35 skills from ESCO and 7 e-competences from EN16234 for SC engineers. SC technicians involve 20 knowledge items and 19 skills from ESCO and 7 e-competences from EN16234. The whole set of information for both profiles with the extended data and analysis from the SMACITE projects allows a proper answer to RQ1 and offers a complete basis for the work of the subsequent research questions presented in the following sections.

## 4. Analysis of the Occupation Profiles in the ESCO Labor Classification Most Similar to the Recommended Qualification Profile for SC Projects

The ESCO labor classification works as a dictionary, describing, identifying, and classifying professional occupations and skills relevant for the EU labor market. Version 1.2 provides descriptions of 3039 occupations and 13,939 skills linked to these occupations, translated into all official EU languages. It is available in an online portal (https://esco.ec.europa.eu/en, accessed on 15 July 2024) and can be consulted free of charge. More than 200 experts developed it over more than 4 years, and it has been the compulsory reference for labor classification in all member states of the European Union since 2021.

The consultation of data from the ESCO classification is not effective with the browsing functionality offered on the website, as the interface does not allow sophisticated queries to provide relevant information in a feasible and efficient way. ESCO provides a way to download or retrieve its basic constituent elements, such as a list of occupations, skills, relations among elements, etc. Using these items, we could reconstruct the ESCO database to develop meaningful queries for exploring occupation profiles.

The ESCO classification does not have any equivalent or very similar occupation profile referring to SC projects; moreover, even the term Smart City does not explicitly appear in any occupation. There is only a general knowledge item named “Smart Cities features,” which is mentioned as essential for the occupation “mobility services manager” and as optional for “urban planner” and for “autonomous driving specialist.” Therefore, we explored the similarity of occupation profiles in professionals and technicians in engineering and the IT profession with the recommended profile for SC engineers and technicians. The goal was the determination of the occupations most similar to the profiles requested by SC projects, which could suggest which existing ESCO occupations could be more easily adapted or trained to the work in SC.

The process started with the 89 skills previously determined for reference in the survey of Section 3, together with their allocation to each of the main technical and non-technical areas recommended for the SC profiles, as follows: Internet of Things, cybersecurity, cloud computing, data analytics and visualizations, machine learning and big data, project and business management, and green/environment. Using the locally reconstructed database and queries through the 123,372 relations between the 3039 occupations and 13,939 skills and knowledge items, the exploration was focused on the following groups of occupations of ESCO that correspond to the SC profiles:

- SC engineers:
  ○ A total of 1223 research and development managers;
  ○ A total of 1330 information and communications technology service managers;
  ○ A total of 21 science and engineering professionals;
  ○ A total of 25 information and communications technology professionals.
- SC technicians:
  ○ A total of 31 science and engineering associate professionals;
  ○ A total of 35 information and communications technicians.

After performing queries and analyzing the resulting data (2710 records), the results of the similarity between ESCO occupations and SC project profiles are shown in Table 3 for the SC engineers and Table 4 for the SC technicians. The occupations are specified with the ISCO code for their group of occupations (https://ilostat.ilo.org/methods/concepts-and-definitions/classification-occupation/, accessed on 23 July 2024). The tables only show the top profiles in mentions to the skills and knowledge items present in their ESCO profiles, as well as the number of skill areas where those skills belong, both in the set of all areas (including non-ICT areas like project and business management and green skills) and those only in ICT areas, in the case of the SC technicians without including machine learning and big data, in correspondence to their profile (see Section 3.2.2).

As can be seen in the results, technical/engineering non-ICT profiles could also be close to the recommended SC profiles when only considering the number of skills. However, as a multidisciplinary field, it is relevant to also see the existing coverage of the different areas, both the global set and the ICT ones. If we consider a combined value of the average coverage in percentage of the three numbers (skills, all areas, and ICT areas), the ranking of similarity changes significantly, as can be seen in Table 5 and Table 6.

It can be surprising to see no specific ICT profiles in the list, but this does not mean that these profiles are automatically qualified to work in the technical profiles for SC projects. The coverage of the recommended skills is far from being complete in all cases, so specific training and skills development would be required. Moreover, the wide multidisciplinary nature of SC projects tends to require different specializations in profiles. Some occupations have a higher percentage of covered skills, while others have better coverage of the different areas. It would be interesting to analyze the adaptability to acquire the rest of the knowledge and skills recommended for the best performance in SC projects. As part of the general reflection on the availability of skills and knowledge of each area in the set of analyzed occupations, Table 7 shows the number of considered profiles for both SC profiles and how many of them have at least one skill in each area and the average number of skills detected in the profiles for each area. This could also be part of the reflections on the need of training for the adaptation to SC projects. As we can see, IoT, machine learning and big data, and cloud computing are the areas with the lowest availability of skills in the profiles. In general, we see the lowest availability of skills in ESCO profiles for technicians.

It would be also possible to compare the existing ICT profiles expressed as EN16234 e-competences (see Section 3.2) with the recommended SC profiles. However, the existing standardized 30 ICT role profiles with e-CF competences (in the annex of the standard EN16234 [41]) offer a limited use for the comparison, as they were explicitly limited to mention only the most important five e-competences. There is additional difficulty, as the similarity must consider both the e-competences and the proficiency level. Unfortunately, none of the standard role profiles match more than one pair of e-competence and level of the SC profiles.

The extensive information in this section on the analysis of similar occupations, in reference to ESCO, provides an answer to RQ2. This is useful for providing insights into the sources of possible specialists for SC projects, as well as depicting a theoretical study of the preliminary needs of training to qualify professionals, according to SC profiles. As a complement, to assess the option of training for the supply of SC technical specialists, the next section will present results of an experience of a training program on the technical areas of the SC profiles within the SMACITE project.

## 5. Analysis of Results of Preliminary Assessment of a Sample of Candidates in a Training Program on SC Profiles

As commented on in the previous section, there are no equivalent profiles to the presented SC engineer and SC technician in the ESCO labor classification. The need for training to adapt qualification to the specific features of SC projects becomes evident to ensure a good availability of technical personnel. While the work in [42] also treated a training program on a set of three SC profiles defined in [22], the available information was extremely focused on the course design and the feedback of the students. The SMACITE project has also designed and implemented a training program oriented to the SC profiles defined and presented in Section 3. While the details of the courses with a separated set of modules for each profile (SC engineer and technician) have been already detailed in [38], it is relevant to mention that the program included a diagnostic tool for the skills and the knowledge of each of the technical areas based on the assessment questions extracted from the module contents, already aligned with the SC profiles. Thus, candidates for the online training programs could better select in advance the modules that could be more useful to them, as they can see how far they are from the desired qualification profile. The details on the tool are also available at [38].

**Figure 6:**
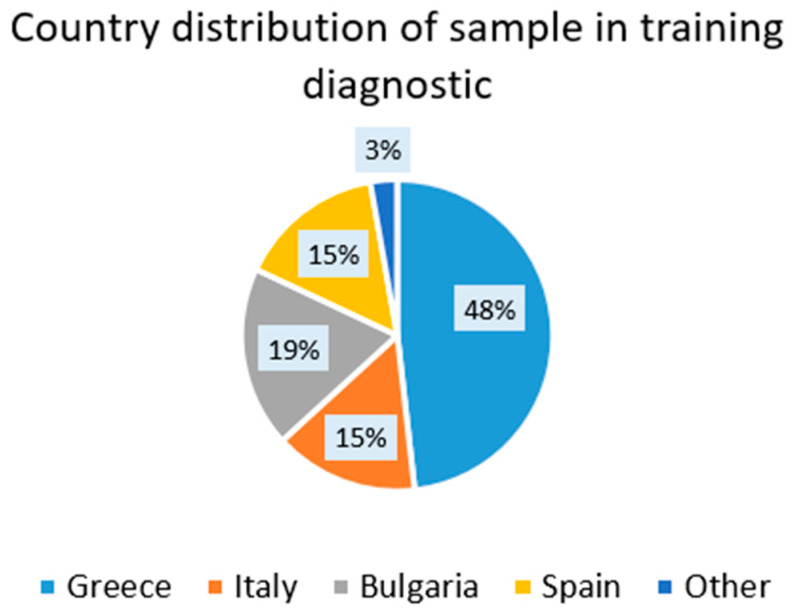
Geographical distribution of the sample of training diagnostic results.

The results of the 256 preliminary assessments from a sample of candidates for the SC engineer and technician modules could provide some insights into the presence of skills and knowledge on the SC areas in interested professionals prior to the exposure to training processes. This could provide an orientation on the degree of familiarity of people with the relevant concepts of the qualification for SC projects in the following six areas: Smart Cities foundations, cloud computing, IoT, cybersecurity, and data analytics (and machine learning for SC engineers). The type of diagnostic assessment in each module is different for the case of the training program for the SC engineer and for the SC technician. The sample of candidates had a varied set of educational backgrounds (see Table 8) with a varied geographical distribution (Figure 6), while the gender distribution was unbalanced (72% of male vs. 28% of female).

The results, as scores of the diagnostics for the skills and knowledge for SC engineers and technicians in each module, are shown in Table 9. As we can see, except for a slightly higher value in data analytics, it is not possible to assume a general knowledge of the technical areas of the involved sample, which is coincidental with the low presence of the skills and knowledge in the technical areas in the existing ESCO profiles (Table 7). The results differ depending on the educational background, although the sample is not large enough to extract clear results by segmenting the data. However, it is possible to suggest that the highest average score in SC engineers’ training pre-assessments corresponds to ICT postgraduates (43.2), followed by other science/engineering postgraduates (24.4), while science/engineering graduates reached 15.4 and ICT graduates reached 12.9. This suggests the importance of specific training on the technical areas associated with the SC profiles to ensure the availability of properly qualified personnel for the forecasted increment of activity and projects linked to SC.

The candidates for the training programs could select the modules that they wanted to participate in and, finally, the number of passed modules in the SC engineers’ branch was 67, while, in the technicians’ one, it was 21.

## 6. Discussion and Results

The aim of this work is to analyze the availability of qualified technical personnel for SC projects, which is the main point analyzed in this research, with a process guided by the three research questions presented in Section 2. The process started with the development of the two main profiles for SC projects (SC engineers and SC technicians), thanks to an extended dataset from a survey collecting information from a varied set of 142 stakeholders. The analysis of data allows us to answer RQ1 and refine the previous analysis of ICT professional profiles restricted to IoT projects [17]. All of the functions related to IoT, data analytics, cybersecurity, cloud computing, project management, and civil engineering, which were top ranked as essential or relevant for SC engineers, are supported by at least 83% of respondents. In the case of the engineers, the most technical functions received lower percentages than the managerial ones, such as project management and civil engineering, with 92% of the responses being essential or relevant. This suggests that the respondents allocate a clear role of management to engineers, although obviously supported by a strong technical background. In the case of SC technicians, the results indicate limited support for the technicians being involved in specific areas such as data analytics, project management, cybersecurity, or cloud computing. Their contribution tends to be more oriented to the support of IoT functions (88% indicated IoT as essential or relevant for SC technicians). All of the functions were ranked as essential or relevant by at least 66% of the surveyed stakeholders, meaning that all of them are part of the SC profiles.

A more detailed analysis was conducted by including 89 specific knowledge and skill items identified in ESCO within the survey. Overall, knowledge tends to be valued more highly than skills for engineers compared to technicians. Non-technical areas, such as business management and environmental sustainability, are perceived as largely irrelevant for technicians, according to the survey respondents, in comparison to engineers. When focusing on SC technicians, new areas such as management and green knowledge and skills received less support, with only 33–37% of experts deeming business-related knowledge and skills essential or relevant, and 43–47% doing so for green knowledge and skills, respectively. Among those surveyed, at least 57.8% highlighted all of the proposed sets of knowledge and skills, including sustainability aspects for the twin transition, as essential or relevant for engineers. The findings reveal that technical knowledge and skills in areas such as IoT, cybersecurity, cloud computing, and data analytics are more widely accepted, with over 74% of respondents considering them essential or relevant for engineers. For Smart City technicians, the alignment between the required knowledge and skills and the necessary functions is evident, with IoT, cybersecurity, and cloud computing being identified as areas more needed in these profiles. The domain of machine learning and big data, encompassing both skills and knowledge, ranks as the least relevant. While approximately 70% of the respondents consider it important for engineers, only around 50% view it as significant for technicians, indicating that this area is not a key factor in the qualification profile of SC technicians.

It is not possible to find any direct or closely related occupational profiles specific to Smart City projects in the ESCO classification, which is why a clear picture of the profile requirements for SC engineers and technicians is needed to answer RQ2. The analysis of the possible profiles that can be more easily adapted to the required work in SC projects is very relevant information to populate the teams for the expected growth of activity. This can be achieved by analyzing the recommended profiles from ESCO, the official labor classification of the EU. The results shown in Section 4 show that the expected theoretical presence of some of the technical skills and knowledge in those candidate profiles is not frequent, mainly for the most technical areas in ICT, while more transversal aspects like project and business management, sustainability, and data analytics have higher coverage. This fact is consistent with the importance given to the technical modules in the two training experiences for SC profiles [38,42]. The set of ESCO profiles with the highest similarity to the recommended ICT profiles for SC represents a catalogue of possible sources of candidates with the lowest need of training for being involved in project teams. This analysis of the ESCO database, with its huge amount of information, was only possible after locally reconstructing it to allow for effective queries.

Although candidates from other occupations can be trained to fulfill the conditions of the SC profiles, there is always a need of specific training. Section 5 analyzes the need of upskilling actual professional candidates interested in SC through the SMACITE project training program. Data from 256 diagnostic assessments before the training show that there is no broad knowledge of technical areas among the sample of candidates. The results vary by educational background, although the sample is too small to extract definitive conclusions. However, the highest average score in Smart City engineers’ training pre-assessments was achieved by the ICT postgraduates (43.2), followed by the other science/engineering postgraduates (24.4). The Science/engineering graduates scored 15.4, while the ICT graduates reached 12.9. This suggests the critical importance of targeted training in the technical areas associated with Smart City profiles to ensure a sufficiently qualified workforce for the anticipated growth in SC-related activities and projects.

## 7. Conclusions and Future Lines

This article has explored one key factor for the success of SC projects identified in maturity models and specific studies: the availability of qualified technical personnel. Based on the information collected thanks to the SMACITE project, this work has analyzed the opinions of a broad spectrum of stakeholders. The answer to RQ1 showed a specific description of the functions, skills, and knowledge recommended for a good qualification of a technical team, which can help to better prepare technical teams for the successful implementation of SC. The fact that these profiles are mapped to ESCO will ensure a better understanding and improved alignment with terminology (compulsory in all member states of the European Union since 2021, thus facilitating the adoption across the continent). The mapping to EN16234 also ensures an enhanced connection to ICT professionalism practices adopted by organizations in Europe, promoting a good understanding of the recommended profiles.

In the case of RQ2, we have seen how the exploration of the existing profiles in the official ESCO labor classification can suggest the professional profiles of those who would require less training to be adapted to the demands of qualification for the two SC profiles. This information could be useful both for employers and for professionals. Employers have a preliminary indication of those profiles who can be more easily retrained for proper work in SC projects. The professionals also have an indication of how much adaptation they would need for SC projects without a need of an extensive comparison of their CV with the SC profiles.

The specialized training is the focus of the study for RQ3 to complete the analysis of the possible options for supplying the necessary number of qualified profiles for SC projects. The data from the diagnostic assessments also implemented within the training program developed by the SMACITE project have provided insights into the expected level of qualification in the different SC technical areas. Although the sample of assessed candidates for training is limited, but varied, the results are coincidental with the data on the theoretical presence of SC skills and knowledge extracted from the ESCO database (see Section 4).

The results have an evident geographical limitation, as the samples were focused on European stakeholders. To overcome this limitation, a new EU-funded project, SMARCO, has already been awarded and is set to start at the beginning of 2025, extending the geographical representations, as well as expanding the dataset for the study. It will also consider rural communities and not only urban communities, more linked to the idea of Smart Cities.

Although the set of the three research questions provides information on an aspect that has not been deeply explored by the literature, despite its importance for the success of SC projects, it is necessary to extend the work to consider more sources of information and larger datasets for a more complete analysis. Apart from a wider geographical representation and a larger amount of data from surveys and other data collection mechanisms, the possibility of analyzing huge datasets from online job advertisements could be useful for refining the conclusions on the requested profiles for SC projects. The use of datasets from the OVATE Cedefop tool (https://www.cedefop.europa.eu/en/tools/skills-online-vacancies, accessed on 19 July 2024)—which collects millions of online job ads from thousands of job portals, with information already linked to the ESCO terminology—is a very interesting option (already used in previous studies [34]). This could be also complemented with more training experiences for SC profiles, where the possible difficulties for learning the different types of candidates could reveal barriers for upskilling, reskilling, or retraining potential new professionals for SC projects.

## Figures and Tables

**Table 1:** Distribution among roles of different categories.

Main Sector (in Bold) and Subsectors	%
**Public sector and authorities (client side)**	**20.42%**
Policy authority or decision maker	5.63%
Municipal city planner or urbanism expert	0%
Municipal technical manager	3.52%
Municipal technician	2.11%
Other	9.15%
**Business sector and providers (supply side)**	**53.52%**
Business manager in IT solutions provider	19.01%
ICT project manager in solutions provider	15.49%
ICT engineer in solutions provider	9.15%
ICT technician in solutions provider	2.82%
Other	7.04%
**Civil society (user side)**	**26.06%**
Expert in Smart Cities (academia, research, education, etc., and out-of-solution providers)	13.38%
Representative of citizens’ association	2.82%
Sociologists or similar specialists in urban life	1.41%
Other	8.45%

**Table 2:** Mapping of functions to the e-competence of EN16234.

Role	e-Competence	Level
Engineer	B.6 (ICT systems engineering)	4
Engineer	E.2 (project and portfolio management)	4
Engineer	A.6 (application design)	3
Engineer	B.4 (solution deployment)	3
Engineer	E.8 (information security management)	4
Engineer	D.7 (data science and analytics)	3
Engineer	B.3 (testing)	3
Technician	E.2 (project and portfolio management)	2
Technician	B.1 (application development)	2
Technician	B.4 (solution deployment)	2
Technician	E.8 (information security management)	3
Technician	D.7 (data science and analytics)	2
Technician	B.4 (solution deployment)	1
Technician	C.1 (user support)	1

**Table 3:** ESCO occupations with mentions to SC engineers’ skills and skill areas in their profile.

ISCO Group	Occ. Code	Occupation Preferred Name	SC Skills (out of 89)	All Areas (out of 7)	SC ICT Areas (out of 5)
2529	2529.7	ICT security engineer	24	5	4
2511	2511.3	data analyst	17	3	3
2529	2529.8	ICT security manager	15	3	3
2512	2512.1	cloud engineer	14	3	3
1321	1321.2.3	operations manager	13	4	2
2511	2511.4	data scientist	13	3	2
2149	2149.9.2	energy systems engineer	12	7	5
2529	2529.1	chief ICT security officer	12	4	3
2529	2529.4	ethical hacker	12	4	3
2151	2151.1.5	power distribution engineer	11	6	4
2141	2141.8	maintenance and repair engineer	11	5	4
2149	2149.9.8	solar energy engineer	10	6	4
2149	2149.9.7	renewable energy engineer	10	6	4
2151	2151.1.1	electric power generation engineer	10	6	4
2151	2151.1	electrical engineer	10	5	3
1330	1330.1	chief data officer	10	4	4
2151	2151.1.3	electromechanical engineer	10	4	3
2529	2529.3	embedded systems security engineer	10	4	3
2511	2511.13	ICT system analyst	9	5	4
2529	2529.2	digital forensics expert	9	3	3
2529	2529.6	ICT security administrator	9	3	3
1330	1330.7	ICT project manager	9	3	2
2529	2529.5	ICT resilience manager	9	2	1

**Table 4:** ESCO occupations with mentions to SC technicians’ skills and skill areas in their profile.

ISCO Group	Occ. Code	Occupation Preferred Name	SC Skills (out of 89)	All Areas (out of 6)	SC ICT Areas (out of 4)
3113	3113.2	hydropower technician	10	5	4
3512	3512.3	ICT security technician	10	3	3
3113	3113.1.2	electromechanical engineering technician	9	3	3
3131	3131.1	offshore renewable energy plant operator	9	3	3
3115	3115.1.7	marine engineering technician	8	4	2
3118	3118.3.10	marine engineering drafter	8	3	2
3114	3114.1	electronics engineering technician	8	7	5
3118	3118.3.7	electromechanical drafter	8	4	3
3114	3114.1.4	instrumentation engineering technician	5	4	3
3511	3511.1	data center operator	4	6	4
3122	3122.3	industrial assembly supervisor	4	5	4

**Table 5:** Ranking of ESCO occupations with top similarity to SC engineer’s profile.

Occ. Code	Occupation Preferred Name	% Skills	% All Areas	SC ICT Areas	Combined Value
2149.9.2	energy systems engineer	13.5%	100.0%	100.0%	71.2%
2529.7	ICT security engineer	27.0%	71.4%	80.0%	59.5%
2151.1.5	power distribution engineer	12.4%	85.7%	80.0%	59.4%
2149.9.8	solar energy engineer	11.2%	85.7%	80.0%	59.0%
2149.9.7	renewable energy engineer	11.2%	85.7%	80.0%	59.0%
2151.1.1	electric power generation engineer	11.2%	85.7%	80.0%	59.0%
2149.9.6	onshore wind energy engineer	7.9%	85.7%	80.0%	57.9%
2141.8	maintenance and repair engineer	12.4%	71.4%	80.0%	54.6%
2511.13	ICT system analyst	10.1%	71.4%	80.0%	53.8%
2521.4	database integrator	7.9%	71.4%	80.0%	53.1%
1330.1	chief data officer	11.2%	57.1%	80.0%	49.5%
1330.1.1	ICT information and knowledge manager	9.0%	57.1%	80.0%	48.7%
2151.1	electrical engineer	11.2%	71.4%	60.0%	47.6%

**Table 6:** Ranking of ESCO occupations with top similarity to SC technicians’ profile.

Occ. Code	Occupation Preferred Name	% Skills	% All Areas	SC ICT Areas	Combined Value
3113.2	hydropower technician	11.2%	85.7%	100.0%	65.7%
3113.1.2	electromechanical engineering technician	10.1%	57.1%	100.0%	55.8%
3131.1	offshore renewable energy plant operator	10.1%	57.1%	75.0%	47.4%
3115.1.7	marine engineering technician	9.0%	57.1%	75.0%	47.0%
3118.3.10	marine engineering drafter	9.0%	57.1%	75.0%	47.0%
3512.3	ICT security technician	11.2%	42.9%	75.0%	43.0%
3114.1	electronics engineering technician	9.0%	42.9%	75.0%	42.3%
3118.3.7	electromechanical drafter	9.0%	42.9%	75.0%	42.3%
3511.1	data center operator	4.5%	42.9%	75.0%	40.8%
3114.1.4	instrumentation engineering technician	5.6%	42.9%	50.0%	32.8%
3512.4	ICT technician	3.4%	28.6%	50.0%	27.3%

**Table 7:** Availability of skills in the ESCO profiles analyzed for similarity to SC profiles.

	SC Engineers (308 ESCO Profiles)		SC Technicians (99 ESCO Profiles)	
Area	% with 1 Skill	Average Num. Skills	% with 1 Skill	Average Num. Skills
Project management and business	83.1%	1.30	57.6%	0.73
Cloud computing	17.9%	0.35	12.1%	0.21
Cybersecurity	32.1%	0.65	27.3%	0.34
Data analytics and visualizations	34.4%	0.66	24.2%	0.44
Machine learning and big data	11.0%	0.20	8.1%	0.23
Internet of Things	9.1%	0.12	7.1%	0.07
Green/environment	31.5%	0.79	32.3%	0.40

**Table 8:** Educational background of the sample.

Background	%
General secondary education	10.2%
Initial high VET training	12.9%
BSc in ICT	12.4%
BSc in non-ICT engineering or science	17.8%
Postgraduate in ICT	18.5%
Postgraduate in non-ICT engineering or science	18.5%
Other	9.6%

**Table 9:** Results of each module in the preliminary diagnostic.

Training Module Diagnostic	SC Engineers (out of 100)	SC Technicians (out of 100)
Smart Cities foundations	27.1	32
Cloud computing	28.3	33
IoT	39.2	34
Cybersecurity	27	34
Data Analytics	49.5	49
Machine learning and big data	35.3	NA

“NA”: Not applicable.

## Data Availability

The data from the SMACITE survey presented in this study are openly available at Zenodo at https://zenodo.org/records/13284424 (accessed on 9 August 2024).
